# Kansas Veterinary Medical Association

**Published:** 1892-04

**Authors:** N. S. Mayo

**Affiliations:** Secretary


*Kansas Veterinary Medical Association.*—The semi-annual meeting of the Kansas Veterinary Medical Association was held in Manhattan on March 10 and 11, 1892. Meeting was called to order by Vice-president Hunter. The following members were present: Drs. Hunter, Cook, Brady, Orr, and Mayo. The following graduate veterinarians were elected to membership, Drs. Nott, Welch, McCassy, Eisenhower and Wattles. Upon invitation the Association visited the State Agricultural College, and were much pleased with the work being done there.

In the evening Dr. Hunter read a paper entitled, "*What* is it?" describing a peculiar case of malignant tumor in the pharynx of a troop horse. The paper was very interesting and evoked considerable discussion. Dr. Mayo followed with the report of an outbreak of "Mad Itch" or "Hydrophobia," so-called in cattle. The discussion which followed drifted to the interesting subject of "Stalk Disease" in cattle. The other essayists being absent, a question box was introduced, which proved very interesting, every one present taking part in the discussion of questions presented.

According to previous notice, Sec. IV. of the code of ethics was stricken out. A committee, consisting of Drs. Welch, Orr and Mayo, was appointed to draw up a fee bill for the use of the members of the association and present it at the next meeting.

The following resolution was adopted:

*Resolved*, That the Kansas Veterinary Medical Association condemns the method of meat inspection as practiced by the Bureau of Animal Industry at Kansas City, where only one graduate of a veterinary college is employed. Fitness for position and not political influence should be the qualifications for appointment. We consider the method now in vogue in Kansas City as detrimental to the veterinary profession and inimical to the proper inspection of meat.

A vote of thanks was extended to the resident veterinarians for their hospitality. The association then adjourded to meet in Topeka the Thursday of State Fair week.

N. S. MAYO, *Secretary.*

